# One - staged reconstruction of bladder exstrophy in male patients: long - term follow-up outcomes

**DOI:** 10.1590/S1677-5538.IBJU.2015.0581

**Published:** 2017

**Authors:** Amilcar Martins Giron, Marcos Figueiredo Mello, Paulo Afonso Carvalho, Paulo Renato Marcelo Moscardi, Roberto Iglesias Lopes, Miguel Srougi

**Affiliations:** 1 Divisão de Urologia do Departamento de Cirurgia, Universidade de São Paulo, SP, Brasil

**Keywords:** Bladder Exstrophy, Reconstructive Surgical Procedures, Male, Patients

## Abstract

**Introduction:**

The surgical correction of bladder exstrophy remains challenging. In our institution, the repair has evolved from a staged repair to one-stage reconstruction. The one-stage reconstruction includes; bladder closure, Cantwell-Ransley neourethroplasty and abdominoplasty using groin flaps, without the need of pelvic ostheotomies. Repair of urinary continence (UC) and vesicoureteral reflux (VUR) is done after development of the infant.

**Objective:**

To present our experience of our modified one-stage reconstruction of bladder exstrophy in male patients.

**Materials and Methods:**

Medical records of male patients submitted to one-stage reconstruction of bladder exstrophy were analyzed retrospectively. Fifteen exstrophy bladder patients with mean age 4.2±7 years were treated at our institution between 1999-2013.

**Results:**

2 5

**Conclusions:**

One-stage reconstruction minimizes the number of surgical procedures required to achieve UC and potentiates bladder-neck function. The advantages of using groin flaps over current techniques for complete repair are the small risk for penile tissue loss and the avoidance of ostheotomies.

## INTRODUCTION

Classic bladder exstrophy is a rare malformation of the genitourinary tract and its incidence is around 1 case to 30.000 to 40.000 live births (1). The surgical management of bladder exstrophy has evolved during the last years, with the standard treatment until the late 1950s being urinary diversion with ureterosigmoidostomy. Afterwards, in 1970s it evolved to a staged repair, with early pelvic ring approximation and abdominal wall, bladder and posterior urethral closure performed as a first stage, followed by second stage neourethroplasty (modified Cantwell-Ransley technique) and finally a bladder neck surgical reinforcement such as Yong-Dees-Leadbetter procedure (2). The modern staged repair (MSRE) technique involves bladder closure shortly after birth, followed by epispadias repair at age 6-12 months and bladder neck reconstruction at age 4-5 years when it is thought that the child can cooperate with attempting continence.

In 1990s, Mitchell introduced the concept of one-stage reconstruction of extrophy, where all the aforementioned procedures were performed as a single surgery comprehensive approach (3). The concept of this approach was to decrease the number of surgical procedures required to achieve continence as well as achieve early bladder neck resistance and bladder cycling (4). This technique presented good results, although it has some drawbacks such as risk of penile tissue loss and necessity of ostheotomies in older children or after failed repair.

Placing the posterior urethra and bladder deep into the pelvis in combination with a tension-free closure and adequate postoperative management prevent complications and are now consensus among pediatric urologists (5). The two well-described techniques: MSRE (6) and one-stage reconstruction of bladder exstrophy advocates the observance of these fundamental principles (4).

In our institution, the repair of bladder exstrophy has evolved from a staged repair to one-stage reconstruction (Table-1). However, we describe our one-stage reconstruction of bladder exstrophy, performed in University of São Paulo, since late 1990s as a single comprehensive surgery that was adapted to our environment since it was common to receive older children with previous failed repairs. In this procedure, we perform bladder closure and positioned it deep in the pelvis, Cantwell-Ransley neourethroplasty and abdominoplasty using groin flaps, without the need of pelvic ostheotomies. Urinary continence (UC) and vesicoureteral reflux (VUR) are addressed later, at toilet training age.

**Table 1:** Treatment of bladder exstrophy in male patients (time table).

Before 1973		1973-1985	1982-2011	1999-2015
Incontinent Urinary Diversion		Colocystoplasty	Modern Staged Repair	One-Stage Reconstruction
	1. Cutaneous uretero colonic conduit		1. Bladder closure + abdominoplasty	1. Cystorrhaphy + cantwell-ramsley neourethroplasty + abdominoplasty using groin flaps
			2. Phalloplasty + urethroplasty	
	2. Tubularization of the exstrophic bladder			
			3. Bladder neck reconstruction (and ureteral reimplantation)	
				2. Bladder neck reconstruction at age 4 to 5 years (and ureteral reimplantation)
	3. Colonostomy closure + proximally anastomosed to the tubularizated bladder			
			4. Bladder augmentation if necessary	
				3. Bladder augmentation if necessary

## OBJECTIVE

To present our experience of our modified one-stage reconstruction of bladder exstrophy in male patients.

## MATERIALS AND METHODS

Medical records of male patients submitted to one-stage reconstruction of bladder exstrophy were analyzed retrospectively. Fifteen exstrophy bladder patients (16 procedures) with mean age 4.2±7 years (45 days to 22 years) were treated at our institution between September 1999 and October 2013. Nine patients were referred to us after previous failed bladder closure elsewhere. Additionally, five patients had undergone other surgical procedures: inguinal herniorrhaphy in two and urinary diversion in three cases (two colonic conduits, one bilateral cutaneous ureterostomy).

At time of bladder exstrophy repair, patients that were referred to us after previous failed bladder closure elsewhere had a mean age of 6.5 years (2 months to 22 years) and children without previous attempts of repair had a mean age of 9 months (6 to 18 months). At time of data analysis, the group with previous surgery had mean age of 17.7 years (6 to 34 yeas) and the naïve surgical group had a mean age of 9.5 years (3 to 18 years).

**Figure 1:**
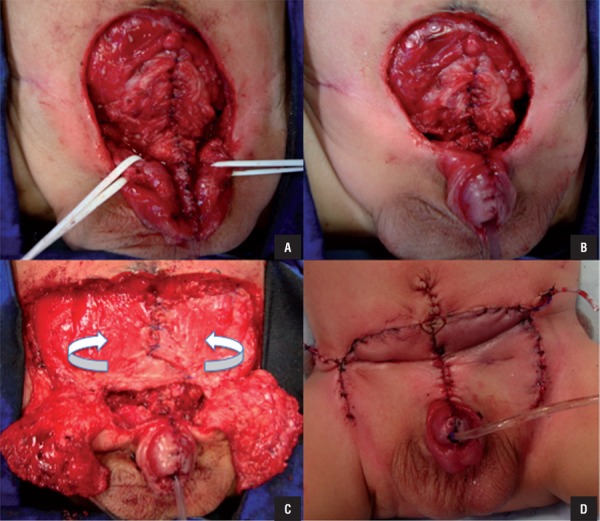
– Steps of one-stage reconstruction: A) Closure of the bladder and tubularized urethra; B) Penile closure; C) Groin flaps; D) Final aspect

All patients were treated with cystorrhaphy, Cantwell-Ransley neourethroplasty, and abdominoplasty using groin flaps to close the abdominal wall defect, without osteotomies. Cystorrhaphy consists in bladder closure in two planes and placing the posterior urethra and bladder deep into the pelvis performing a tension-free closure of the bladder; bladder neck surgery was only performed at time of toilet training. Cantwell-Ransley neourethroplasty begins with extensive dissection of the epispadias but without complete penile disassembly providing easy access to the intersymphyseal ligament, which is deeply incised, dissection of each neurovascular bundle and the urethral plate with its spongiosal tissue, preserving the glans; then the urethral plate is tubularized as shown in Figure-1A. The corpora are rotated medially by approximately 90º and maintained in this new configuration by a proximal caverno-cavernostomy; this new anastomosis between the corpora keeps the urethra in its ventral position and gives the penis a dangling position when flaccid (Figure-1B).

Abdominal wall repair consists in using hypogastric skin and rectus abdominis and obliquus externus abdominis muscle aponeurosis flaps (these groin flaps are rotated to the midline resulting in a very strong abdominal wall). Groin flaps are made of the rectus anterior aponeuroses rotated medially, flipped over, and sutured with prolene sutures to close the defect (Figure-1C) (7). By rotating the facial flaps medially, complete reinforcement of the abdominal wall to the level of the pubic bone is achieved.

All received broad-spectrum intravenous antibiotics (3^rd^ generation cephalosporins) intra-operatively and continued postoperatively (1^st^ generation cephalosporin) and analgesics and anti-inflammatory drugs as needed for 1-2 weeks. A urethral catheter was left for 7-10 days and two plastic catheters were used for drainage of subcutaneous tissue (Figure-1D).

Successful primary closure was defined as an acceptable functional outcome with no wound dehiscence or fistula; failed closure was defined as wound dehiscence.

Considered variables were length of surgery, length of hospital stay, complications related to one-stage reconstruction and urinary continence.

Bladder neck reconstruction was performed at age 4 to 5 years when it is thought that the child can cooperate with attempting continence. Additional procedures were needed in many cases to achieve the treatment goals (urinary continence, normal genital cosmetic and preservation of upper urinary tract). Patients were considered continent if dry after the age of toilet training (3 to 5 years), and for those under this age, if dry for intervals between 2 to 3 hours. They were considered incontinent if any urinary leakage was observed between voiding or catheterizations from either the catheterizable channel or the urethra (for toilet trained children) or if they could not achieve continence intervals of ≥2 hours (for toddlers and infants). Patients who were using diapers were included in the incontinent group.

Regular clinic visits and periodic kidney, urinary tract and bladder ultrasonography were performed every 6 months to 1 year. Voiding cystourethrography was performed only in cases of urinary tract infections, upper urinary tract dilatation and/or urinary incontinence (small intervals with continuous dribbling for infants and toddlers). Urodynamics were performed in patients before bladder neck repair. Bladder cystometric capacity expected to age (BcapE) was determined by Koff’s formula, [(age in years + 2) x 30] mL, for children over 2 years; (7 x body weight in kg) mL, for boys under 2 years old. Augmentation was indicated when bladder capacity was low and/or compliance was poor.

## RESULTS

Mean operative time was 325±61.3 minutes (240 to 420 min) and mean hospital stay was 13.2±5.8 days (6 to 23 days). Successful closure was achieved in 14 patients (93.3%) performing a single procedure; one patient had complete wound dehiscence and needed another reconstruction (6.7%) - this patient had previous bladder closure elsewhere. None had ischemic loss of the glans or corporal bodies. Other four patients (26.6%) presented small fistulas and one (6.7%) presented penile rotation as a complication related to one-stage reconstruction. Mean follow-up was 10.3±4.5 years (2y8mos to 16y).

Nine patients (60%) are continent at present: seven voids spontaneously and two are on clean intermittent catheterization. From the group with previous bladder closure elsewhere, five (55%) achieved continence and from the group of naive patients, four (66.7%) are continent. All patients have normal kidneys on US and normal serum creatinine. Seven patients (46.6%) showed vesicoureteral reflux on voiding cystourethrography performed after urinary tract infections.

Additional procedures were needed to achieve the treatment goals, including upper urinary tract protection, urinary continence and satisfactory cosmetic results. Eleven patients (73.3%) patients underwent bladder neck surgery (nine pts. underwent Young Dees Leadbetter and two was submitted to bladder neck injection of Durasphere), and seven out of eleven achieved continence. Five (33.3%) required augmentation ileocystoplasty and Mitrofanoff stoma to facilitate CIC. There are two out of five patients with bladder augmentation that are still incontinent. VUR needed subsequent treatment in three cases (20%) (ureteral reimplantation). Three boys (20%) required inguinal herniorrhaphy during follow-up (two unilateral and one bilateral). A mean of 3±1.1 procedures (2-5) was accomplished per children. Six patients have still pending procedures (three awaits for augmentation cystoplasty associated with bladder neck reconstruction and three cases need urinary fistula repair) (Table-2).

**Table 2:** Patient characteristics and results of operation with one-stage reconstruction.

Treatment of naive patients						

AA	AR	OT(min)	CS	Aug	ARS	Results
3 years	1.5m	315				Continent (VS)
6 years	2m	310				Incontinent
7 years	4m	315				Incontinent
12 years	1.5m	330	+			Continent (VS)
14 years	10m	240	+	+		Continent (CIC)
15 years	6m	270	+			Continent (VS)

Previous bladder clousure elsewere						

AA	AR	OT(min)	CS	Aug	ARS	Results

7 years	1yr5m	405	+	+	+	Incontinent
11 years	8yr	420				Continent (VS)
12 years	3yr	300	+		+	Incontinent
15 years	11m	265	+	+		Incontinent
16 years	5m	390	+			Continent (VS)
16 years	7m	285	+			Incontinent
19 years	3yr	260	+	+	+	Continent (CIC)
30 years	21yr	350	+	+		Continent (VS)
34 years	22yr	420	+			Continent (VS)

AA = age at analysis; PBC = previous bladder closure; AR = Age at reconstruction; OT = Operative time; CS = Continence Surgery, Aug = augmentation; ARS = Anti-reflux surgery; CIC = clean intermittent catheterization; VS = voids spontaneously.

Patients that were referred to us after previous failed bladder closure elsewhere needed more procedures then children without previous bladder closure, (mean of 3.3±1.1; mean of 2.5±0.8 procedures per children, respectively). The most common additional procedure was bladder neck surgery. Eight patients (88%) from the group with previous bladder closure performed elsewhere needed the procedure, while three patients (50%) from the naive treatment group needed this procedure. Moreover, at present, five out of nine (55%) patients with previous failed bladder closure are continent and four out of six (67%) of those without previous bladder closure are continent.

## DISCUSSION

The management of children with bladder exstrophy remains a challenge and despite the choice of approach (staged versus one-stage reconstruction), patients have to undergo several procedures in order to attain goals of surgical treatment such as: urinary continence, preservation of upper urinary tract and genital function and cosmesis.

Stjernqvist et al. showed a median of 12 procedures to achieve good results in bladder exstrophy staged approach (MSRE) (8). There are few experiences with one-stage reconstruction: Gargollo et al. had a mean of 4 (range 1 to 31) procedures to achieve satisfactory results (9). Ebert et al. reported mean of 2.95 (range 1 to 8) surgeries in patients who underwent single stage repair, with only 13.6% requiring more than 4 surgeries, about half of these patients were referred after failed reconstruction elsewhere (10). In our series, around 73.3% (11 out of 15) of patients were referred after failed reconstruction, and a mean of 3±1.1 procedures (range 2-5) per children was observed, and we still have six patients waiting for additional surgeries.

The incidence of urinary continence after bladder exstrophy repair is variable (12% to 83%) (11-15). Various factors interfere with results analysis. There is no standard definition for continence and as a result studies address continence in a non-uniform way. Patient age at bladder closure, the type of closure performed, the number and type of procedures required to establish continence, the need for concomitant bladder augmentation and the need for clean intermittent catheterization is not reported in most papers. Again, there are few cases treated with one-stage reconstruction. Mitchell and colleagues showed 74% (17 of 23 patients) of daytime continence. Overall, 2 of 10 boys (20%) with bladder exstrophy achieved primary daytime continence with one-stage reconstruction alone and without the need for further bladder neck reconstruction (16). In parallel, we showed 60% of our cohort (9 of 15 patients) continent, but only two (13.3%) achieved continence with one-stage reconstruction alone. This result indicates that one-stage reconstruction alone was mostly not able to give continence and bladder neck surgery is usually necessary.

The incidence of progressive or severe hydronephrosis and/or renal scarring ranges from 0% to 30% after one-stage reconstruction. Later surgical repair of vesicoureteral reflux was necessary in 0-50% of patients (17). In our series, three (20%) patients developed vesicoureteral reflux that required treatment.

The limitations of our study are that the experience with one-stage reconstruction was relatively small and not all patients underwent complete treatment to evaluate the efficacy of this procedure. However, we do believe it has advantages over the traditional approaches to bladder exstrophy. Based in our experience, one-staged reconstruction without osteotomy is feasible at any age, even after previous failed procedures, reducing the surgical steps and facilitating closure of the structures. It helps minimize the total number of surgeries. Improved urethral resistance may increase bladder capacity in young patients and restores bladder cycling, which results in the expansion of even very small reconstructed bladders with poor bladder plates (18, 19). While increased outlet resistance may allow for an increase in bladder capacity, a recognized consequence is elevated bladder pressure, which may lead to upper tract changes. It enables concomitant abdominoplasty, with good cosmetic results. Primary or secondary bladder neck reconstruction is required for optimal continence. In our pool of patients treated with primary repair, the need for bladder augmentation is still significant, but complications are less frequent than in the staged procedures.

Although there is ongoing discussion with regards to achieving continence in exstrophy patients, a successful primary bladder closure, regardless of the use of osteotomy or type of repair undertaken, has been shown to be the single most important predictor of eventual continence (20). Pelvic osteotomy remains to have a role in the surgical management of the exstrophy-epispadias complex, as it decreases tension across the abdominal wall, reduces the pubic diastasis, and helps restore the pelvic ring and floor to the normal anatomical configuration. However, in our series, it was not necessary to perform pelvic ostheotomies and bone mobilization, as we opted to use groin flaps for the abdominoplasty. It is necessary to wait for rectus anterior sheets consistency, which occurs usually after 45-60 days of life. The main reason for us to adopt this approach was that most of our patients were referred after failed attempt and these older children are less amenable to collaborate with traction needed after ostheotomies.

Another advantage of our technique in comparison to Mitchell’s one-stage reconstruction is that we do not perform complete penile disassembly, reducing risks of penile ischemia and loss (Figure-1). In our series, ischemic loss of the glans or corporal bodies was not observed.

## CONCLUSIONS

Most patients with bladder exstrophy will require multiple operations to achieve normal voiding and provide cosmetically acceptable and functional genitalia. One-stage reconstruction minimizes the number of surgical procedures required to achieve the treatment goals (urinary continence, normal genital cosmesis and preservation of upper urinary tract). The advantages of using groin flaps over current techniques for complete repair are the small risk for penile tissue loss and the avoidance of pelvic ostheotomies. The major drawback of this technique is the necessity to correct the bladder exstrophy defect after 45-60 days of life (wait to rectus anterior sheets consistency) and also a theoretical risk of malperfusion and loss of flaps, which was not observed in this study.

## ARTICLE INFO

Int Braz J Urol. 2017; 43: 155-62
